# The role of experience level in radiographic evaluation of femoroacetabular impingement and acetabular dysplasia

**DOI:** 10.1093/jhps/hnu005

**Published:** 2014-08-22

**Authors:** Patrick C. Schottel, Caroline Park, Anthony Chang, Zakary Knutson, Anil S. Ranawat

**Affiliations:** 1. Department of Orthopaedic Surgery, Hospital for Special Surgery, 535 East 70^th^ St, New York, NY 10021, USA; 2. Department of Radiology, Sharp Rees-Stealy Medical Center, San Diego, CA, USA; 3. Department of Orthopaedic Surgery, Bone and Joint Hospital at St. Anthony, Norman, OK 73072, USA

## Abstract

Accurate radiographic interpretation is essential for properly diagnosing the etiology of pre-arthritic hip pain such as femoroacetabular impingement (FAI) and acetabular dysplasia (AD); however, radiographic interpretation can be significantly influenced by the observer’s experience level. This study assesses the accuracy and inter- and intraobserver reliability in the radiographic evaluation of FAI and AD based on experience level. Fifty-five patients diagnosed with FAI, AD or normal hip morphology were identified from the principal investigator’s institutional database. Four observers performed an independent and blinded radiographic review, assessing 14 radiographic parameters and an interpretation of a final diagnosis. A second radiographic evaluation of 20 preselected cases was completed 6 weeks after the initial reading to assess intraobserver reliability. Inter- and intraobserver reliability was determined using Cohen’s Kappa Coefficient (*κ*) and intraclass correlation coefficient (ICC) for continuous parameters in a four-rater design. Interobserver reliability was highest across experience levels for lateral centre edge angle (ICC = 0.92) and alpha angle (ICC = 0.90) and lowest (*κ* < 0.3, ICC < 0.3) for joint congruency and detection of herniation pits. Intraobserver reliability was highest for acetabular depth (*κ* = 0.89) and alpha angle (ICC = 0.80) and lowest for head–neck offset ratio and Tönnis grade. Final diagnosis was consistent with the original blinded clinical diagnosis 75–84% of the time across four experience levels. The attending orthopaedic hip surgeon demonstrated greatest diagnostic sensitivity but lowest specificity for making an accurate radiographic diagnosis. Subjective parameters must be redefined, and objective parameters must be further developed to improve the reliability of accurately diagnosing FAI or AD.

## INTRODUCTION

Pre-arthritic hip conditions such as femoroacetabular impingement (FAI) and acetabular dysplasia (AD) have been recognized to predispose patients to early hip osteoarthritis (OA) [1–6]. A delay in diagnosis for these patients could preclude them from pursuing a timely joint preserving surgery and instead result in worsening joint degeneration requiring replacement [7]. Unfortunately, FAI and AD often have overlapping symptoms, making an accurate diagnosis challenging. Therefore, radiographs remain the cornerstone in the evaluation, diagnosis and management of patients with these pre-arthritic hip conditions [8, 9]. The ability to accurately and consistently interpret the radiographs in this particular patient population is of great importance.

There are a number of radiographic parameters used to measure and describe AD as well as FAI; however, it is unclear which of these parameters are considered the most useful for an accurate diagnosis [8]. Several studies to date have examined the interobserver and intraobserver reliability of individual radiographic measurements such as alpha angle, head sphericity, head–neck offset and acetabular index [10–15]. However, the majority of these publications have focused exclusively on measuring the reliability amongst only orthopaedic hip specialists [16]. Since patients are often seen by physicians of varying levels of experience, dependable radiographic parameters to assess FAI and AD should yield the same assessment after multiple readings by different observers regardless of their experience level. Additionally, no study to our knowledge has looked to see if increasing surgical experience leads to a higher tendency of ‘over-reading’ radiographs, thereby resulting in a greater number of pathologic diagnoses and potential recommendation for further work-up or surgical intervention [17].

The objective of this study was to determine the interobserver and intraobserver reliability for the radiographic evaluation and diagnosis of FAI and AD between physicians with varying levels of experience. Additionally, we were interested in analysing the final radiographic diagnosis to investigate whether there was a tendency for more experienced surgeons to read radiographs as abnormal. Our hypothesis was (i) there will be poor interobserver reliability across experience levels, (ii) there will be better intraobserver reliability with more experienced readers and (iii) more experienced surgeon readers will categorize a greater number of radiographs as being abnormal.

## MATERIALS AND METHODS

After Institutional Review Board approval, a statistician from the author’s institution determined the number of cases needed for each group to determine statistical significance in regard to interobserver and intraobserver reliability for each radiographic parameter and final diagnosis.

Fifty-five patients were then identified from the principal investigator’s institutional patient database. ICD-9 codes were initially used to identify patients with FAI, AD and normal hip morphology. Anteroposterior (AP) and elongated neck lateral radiographs for 20 patients with FAI (cam or pincer morphology), 20 patients with AD and a control group of 15 patients without abnormal hip morphology were selected. All FAI and AD subjects were greater than 18 years of age with a confirmed clinical diagnosis based on clinical, radiographic and surgical findings. Controls were required to have been evaluated clinically and radiographically by their surgeon and found to have no evidence of hip disease or previous hip surgery. Finally, all radiographs were standardized by evaluating for excessive rotation and tilt using the gender neutral symphysis-sacrococcygeal joint distance as described by Sibenrock *et al*. [18]. Patients with excessively tilted or rotated AP radiographs were excluded.

De-identified AP and elongated neck lateral radiographs were taken from our institution’s picture achieving and communication system (PACS) for each study patient and individually stored on a compact disc. All images retained the capacity to be manipulated as needed by each reader with use of the bundled PACS measurement tools. Case order was randomly assigned for each of the three diagnoses.

Four observers from the same institution—an attending orthopaedic hip surgeon, an attending musculoskeletal radiologist, an orthopaedic sports fellow and a third-year orthopaedic surgery resident—performed a blinded radiographic review of all 55 patients. Observers assessed 14 radiographic parameters and made a final diagnosis of FAI, AD or normal.

The objective radiographic parameters for this study included acetabular inclination, alpha angle (lateral), head–neck offset, head–neck offset ratio, head–neck offset ratio (lateral) and lateral centre edge angle. Subjective radiograph features included acetabular depth, acetabular version, detection of herniation pits, head sphericity, head sphericity (lateral), joint congruency, position of head centre and Tönnis grade. A second radiographic evaluation of 20 randomly selected cases was completed 6 weeks after the initial reading to assess intraobserver reliability.

## STATISTICS

Intraobserver and interobserver reliability was determined using Cohen’s Kappa coefficient (*κ*) and intraclass correlation coefficient (ICC) as well as the corresponding 95% confidence intervals for continuous parameters in a four-rater design. For intraobserver reliability, agreement was calculated for each observer separately. The pooled estimates of the intraobserver Kappa statistics/ICC, the corresponding asymptotic standard errors and the 95% confidence intervals were also determined. All statistical analysis was performed using SAS 9.2 for Windows (SAS, Cary, NC).

## RESULTS

Interobserver reliability was highest across all experience levels for lateral centre edge angle and alpha angle (ICC = 0.92 and ICC = 0.90, respectively) and lowest for acetabular depth, joint congruency, Tönnis grade and detection of herniation pits. The agreement between all readers for final diagnosis was moderately reliable (ICC = 0.68; range 0.60–0.75) (Table I) [19]. Of all 14 radiographic parameters, seven demonstrated poor interobserver reliability with kappa or ICC values less than or equal to 0.40 (Table I) [18]. Eighty-six percent (6 of 7) of these unreliable parameters were considered to be subjective.

**Table I.** Interobserver reliability

	Interobserver reliability		
	Four raters		
	Kappa/ICC *N* = 55	95% CI	
*Objective parameters*			
Acetabular inclination (w)	0.43	0.35	0.51
Alpha angle lateral (i)	0.90	0.86	0.92
Head–neck offset AP (w)	0.49	0.41	0.57
Head–neck offset ratio AP (i)	0.37	0.23	0.49
Head–neck offset lateral (w)	0.44	0.36	0.51
Lateral centre edge angle AP (i)	0.92	0.90	0.94
*Subjective parameters*			
Acetabular depth (s)	0.23	0.13	0.34
Acetabular version (s)	0.40	0.29	0.51
Herniation pits AP (s)	0.16	0.05	0.27
Head sphericity AP (s)	0.38	0.27	0.49
Head sphericity lateral (s)	0.44	0.34	0.55
Joint congruency (s)	0.02	–0.09	0.13
Position of head centre (s)	0.49	0.38	0.60
Tönnis grade (w)	0.22	0.12	0.31
Diagnosis (w)	0.68	0.60	0.75

s, Simple kappa coefficient (95% CI); w, weighted kappa coefficient (95% CI); i, ICC (95% CI).

Intraobserver reliability was highest for acetabular depth and alpha angle (*κ* = 0.89 and ICC = 0.80, respectively). The head–neck offset ratio (ICC = 0.38) had the poorest reliability amongst the individual readers (Table II). For the final radiographic diagnosis, intraobserver reliability ranged from moderate to excellent across the various readers (*κ* = 0.54–1.0). The pooled intraobserver reliability for all readers was considered to be excellent (ICC = 0.79) (Table II) [18].

**Table II.** Intraobserver reliability

	Intra-observer reliability				
Variable	Rater 1 Fellow *N* = 20	Rater 2 Radiologist *N* = 20	Rater 3 Resident *N* = 20	Rater 4 Attending *N* = 20	Pooled estimates (All readers)
*Objective parameters*					
Acetabular inclination (w)	0.88	0.62	1	0.55	0.76
	(0.71, 1.00)	(0.33, 0.91)	(1, 1)	(0.28, 0.82)	(0.55, 0.98)
Alpha angle lateral (i)	0.88	0.81	0.71	0.79	0.80
	(0.72, 0.95)	(0.59, 0.92)	(0.41, 0.87)	(0.55, 0.91)	(0.56, 1)
Head–neck offset AP (w)	0.53	0.64	1	0.58	0.69
	(0.19, 0.88)	(0.19, 1)	(1, 1)	(0.23, 0.93)	(0.36, 1)
Head–neck offset ratio AP (i)	0.41	0.60	0.10	0.42	0.38
	(−0.02, 0.71)	(0.24, 0.82)	(−0.34, 0.51)	(−0.01, 0.72)	(−0.03, 0.8)
Head–neck offset lateral (w)	0.45	0.67	0.59	0.71	0.61
	(0.08, 0.82)	(0.33, 1)	(0.27, 0.92)	(0.47, 0.95)	(0.28, 0.93)
Lateral centre edge angle AP (i)	0.95	0.88	0.79	0.36	0.75
	(0.88, 0.98)	(0.74, 0.95)	(0.56, 0.91)	(−0.07, 0.68)	(0.49, 1)
*Subjective parameters*					
Acetabular depth (s)	1	1	1	0.57	0.89
	(1, 1)	(1, 1)	(1, 1)	(0.14, 1)	(0.68, 1)
Acetabular version (s)	0.60	0.58	0.38	0.58	0.54
	(0.26, 0.94)	(0.19, 0.98)	(−0.09, 0.86)	(0.24, 0.91)	(0.15, 0.92)
Herniation pits AP (s)	0.42	−0.05	1	1	0.59
	(0.06, 0.78)	(−0.12, 0.02)	(1, 1)	(1, 1)	(0.41, 0.78)
Head sphericity AP (s)	0.48	0.86	0.10	0.71	0.54
	(0.11, 0.85)	(0.59, 1)	(−0.28, 0.48)	(0.41, 1.00)	(0.20, 0.87)
Head sphericity lateral (s)	0.70	0.68	0.60	0.29	0.57
	(0.40, 1.00)	(0.35, 1)	(0.28, 0.92)	(−0.16, 0.74)	(0.21, 0.92)
Joint congruency (s)	0.12	1	1	0.63	0.69
	(−0.32, 0.56)	(1, 1)	(1, 1)	(0.24, 1.00)	(0.39, 0.98)
Position of head centre (s)	0.70	0.30	0.39	0.78	0.54
	(0.40, 1.00)	(−0.10, 0.70)	(−0.01, 0.80)	(0.50, 1.00)	(0.19, 0.89)
Tönnis grade (w)	0.44	1	0.17	0.31	0.48
	(0.15, 0.72)	(1, 1)	(−0.05, 0.39)	(−0.19, 0.81)	(0.17, 0.79)
Diagnosis (w)	0.92	1	0.54	0.68	0.79
	(0.78, 1.00)	(1, 1)	(0.19, 0.89)	(0.39, 0.96)	(0.55, 1)

s, simple kappa coefficient (95% CI); w, weighted kappa coefficient (95% CI); i, ICC (95% CI).

Finally, we found that surgical experience had an effect on the final radiographic diagnosis. The attending orthopaedic hip surgeon demonstrated the highest sensitivity (90%) but lowest specificity (53.3%) in making an accurate diagnosis. Of his incorrect diagnoses, 63.6% (7 of 11) were because of ‘over-calling’ the case by identifying a pathologic condition in a normal radiograph. The orthopaedic sports fellow had a slightly lower diagnostic sensitivity of 82.5% and the junior orthopaedic resident demonstrated the lowest sensitivity (75%) but highest specificity (86.6%). Of the resident’s incorrect diagnoses, only 16.6% (2 of 12) were due to falsely identifying a pathologic condition. The difference in the rate of false positives between the attending orthopaedic surgeon and resident readers was statistically significant (*P* = 0.036).

## DISCUSSION AND CONCLUSION

Radiographs remain the cornerstone in the diagnosis and management of pre-arthritic hip conditions such as FAI and AD. Accurate radiographic evaluation is of paramount importance as these conditions can often have overlapping clinical symptoms. However, radiographic interpretation of subtle abnormal hip morphology can be difficult, particularly to the untrained eye. For this study, we wanted to see how a varying level of experience affects intraobserver and interobserver reliability. Based on our statistical analysis of a four-rater system composed of physicians with a broad spectrum of clinical experience, we found a relatively low level of interobserver and intraobserver reliability between readers, especially for subjective parameters. Thus, many of the standard radiographic measurements on AP pelvis and lateral hip views to diagnose FAI or AD were observed as not being reproducible.

We found interobserver reliability to be highest among objective parameters such as the lateral centre edge angle and alpha angle. Poor interobserver reliability values were observed with predominately subjective measures such as joint congruency, Tönnis grade and detection of herniation pits. In fact, of the radiographic markers that were found to have poor interobserver reliability, six of the seven were subjective parameters. Our findings are similar to two other studies that have also looked at the reliability of pre-arthritic radiographic hip parameters [16, 20]. Carlisle *et al.* [16] assessed reliability using observers with a narrower range of surgical hip experience—two orthopaedic residents, one orthopaedic adult reconstruction fellow, one sports orthopaedic attending without interest in the hip and two attending musculoskeletal physiatrists. Similar to our findings, they observed that the centre edge angle was the most reliable interobserver measurement (ICC = 0.64) and that, in general, the more objective radiographic parameters were more reliable [16]. Another study by Clohisy *et al*. examined the reliability of the same 14 radiographic parameters as our study. In contrast, they employed a rater system composed of only hip specialists—one orthopaedic hip fellow and five orthopaedic attendings all with extensive hip experience. They found the highest interobserver reliability with acetabular inclination, position of head centre and Tönnis grade [20]. Interestingly, all three of these parameters had kappa values lower than 0.5 in our study.

Intraobserver reliability was highest in our study for acetabular depth, alpha angle, acetabular inclination and lateral centre edge angle. Additionally, we observed that intraobserver reliability was generally higher than interobserver values. These findings support similar observations made in prior publications. Carlisle *et al.* [16] found excellent intraobserver reliability for all radiographic parameters including lateral centre edge angle, Tönnis angle, head– neck offset with a frog-leg and cross-table lateral as well as the alpha angle with a frog-leg and cross-table lateral. Only the Tönnis grade had poor reliability [16]. In contrast, Clohisy *et al.* found that only acetabular inclination, position of femoral head and acetabular depth had intraobserver reliability kappa values >0.60 [20]. We had similar findings of high reliability with two of these three measurements (acetabular inclination and depth). Taking our results as well as those of the two aforementioned studies, we could not find one single radiographic parameter that demonstrated consistently excellent interobserver and intraobserver reliability across all studies.

One unique aspect of our investigation was that we found that the amount of surgical hip experience increased the likelihood of radiographically making a pathologic diagnosis. Our senior hip surgeon demonstrated the highest diagnostic sensitivity among all readers. In other words, he most often correctly diagnosed a radiograph as abnormal (90.0%) than the orthopaedic sports fellow (82.5%) or resident (75.0%). However, he also demonstrated the lowest degree of diagnostic specificity meaning he was most likely to diagnosis a normal radiograph as abnormal. Being a high sensitivity but low specificity evaluator is clinically beneficial as it serves as an effective screening tool in identifying the greatest number of individuals with a pathologic condition so that further diagnostic workup (e.g. CT and MRI) or treatment (physical therapy, diagnostic cortisone injection, etc) can be initiated. This also has important implications for the increasing number of hip arthroscopies being performed every year [17]. The tendency to overdiagnose radiographs can explain the increasing incidence of hip arthroscopy procedures. Clear surgical indications should depend on radiographs in conjunction with advanced imaging (CT and MRI), physical exam findings and hip injections. Advanced imaging is especially important in the assessment of FAI and AD as modalities such as three-dimensional (3D) CT have been shown to be as accurate as radiographs for diagnosing and characterizing hip pathologies such as FAI and AD [21, 22, 23]. Advances in imaging will continue to improve our diagnostic ability in evaluating the pre-arthritic hip; however, the cornerstone of FAI and AD diagnosis and evaluation of treatment is properly performed radiographs.

Our study was not without limitations. Since only radiographs were evaluated, observers could not use clinical information such as patient history or physical examination findings to help corroborate any abnormalities seen on radiograph. This could be seen as a possible detractor in the clinician’s ability to determine the final diagnosis and emphasizes the importance of a complete clinical work-up and the need to use other imaging modalities to confirm radiographic findings before proceeding with treatment. Additionally, although an attempt was made to standardize the AP pelvis radiographs by recognizing cases with extreme tilt or rotation, the images were not corrected using computer-assisted methods that have been described in the literature [14, 24]. Because of this there is a possibility that subtle rotation and tilt of the pelvis on AP radiographs inappropriately influenced the physician’s interpretation of radiographic features such as acetabular retroversion.

In conclusion, we found that objective radiographic measurements such as lateral centre edge angle and alpha angle had stronger interobserver and intraobserver reliability than more subjective measurements such as Tönnis grade. We believe that radiographic evaluation ideally allows for consistent assessment between observers to come to the same conclusion on multiple readings regardless of varying skill set or specialty. Therefore, we believe that subjective radiographic parameters need to be redefined and objective parameters need to be further developed to improve the reliability of accurately diagnosing FAI or AD. Additionally, we found that surgical experience not only increased the sensitivity of diagnosing a pathologic condition based on radiograph but also led to a higher rate of ‘over-reading’ the radiograph and making a falsely positive diagnosis.

## SOURCE OF FUNDING

No external funding source.

## CONFLICT OF INTEREST STATEMENT

None declared.

## References

[1] Stulberg SD Acetabular dysplasia and the development of osteoarthritis of the hip. In: Harris WH, (ed.). The Hip: Proceedings of the Second Open Scientific Session of the Hip Society. St Louis, MO: CV Mosby, 1974, 82–93.

[2] Stulberg SD Unrecognized childhood hip disease: a major cause of idiopathic osteoarthritis of the hip. In: Cordell LD Harris WH Ramsey PL MacEwen GD, (eds). The Hip: Proceedings of the Third Open Scientific Meeting of the Hip Society. St Louis, MO: CV Mosby, 1975, 212–28.

[3] Cooperman DR Wallensten R Stulberg SD Acetabular dysplasia in the adult. Clin Orthop Relat Res 1983; 175: 79–85. 6839611

[4] Murphy SB Ganz Muller ME The prognosis in untreated dysplasia of the hip. A study of radiographic factors that predict the outcome. JBJS Am 1995; 77: 985–89. 7608241 10.2106/00004623-199507000-00002

[5] Wilson AS Cui Q Current concepts in management of femoroacetabular impingement. World J Orthop 2012; 3: 204–11. 23362464 10.5312/wjo.v3.i12.204 PMC3557322

[6] Hasegawa Y Iwata H Mizuno M The natural course of osteoarthritis of the hip due to subluxation or acetabular dysplasia. Arch Orthop Trauma Surg 1992; 111: 187–91. 1622705 10.1007/BF00571474

[7] Troelsen A Assessment of adult hip dysplasia and the outcome of surgical treatment. Dan Med J 2012; 559: 1–17. 22677250

[8] Ranawat AS Schulz B Baumbach SE Radiographic predictors of hip pain in femoroacetabular impingement. HSS J 2011; 7: 115–19. 22754409 10.1007/s11420-010-9192-x PMC3145863

[9] Peelle MW Della Rocca GJ Maloney WJ Acetabular and femoral radiographic abnormalities associated with labral tears. Clin Orthop Relat Res 2005; 441: 327–33. 16331022 10.1097/01.blo.0000181147.86058.74

[10] Clohisy JC Nunley RM Otto RJ The frog-leg lateral radiograph accurately visualized hip cam impingement abnormalities. Clin Orthop Relat Res 2007; 462: 115–21. 17589367 10.1097/BLO.0b013e3180f60b53

[11] Gosvig KK Jacobsen S Palm H A new radiological index for assessing asphericity of the femoral head in cam impingement. J Bone Joint Surg Br 2007; 89: 1309–16. 17957069 10.1302/0301-620X.89B10.19405

[12] Meyer DC Beck M Ellis T Comparison of six radiographic projections to assess femoral head/neck asphericity. Clin Orthop Relat Res 2006; 445: 181–5. 16456309 10.1097/01.blo.0000201168.72388.24

[13] Lohan DG Seeger LL Motamedi K Cam-type femoral-acetabular impingement: is the alpha angle the best MR arthrography has to offer? Skeletal Radiol 2009; 38: 855–62. 19565238 10.1007/s00256-009-0745-3

[14] Tannast M Mistry S Steppacher SD Radiographic analysis of femoroacetabular impingement with Hip2Norm-reliable and validated. J Orthop Res 2008; 26: 1199–205. 18404737 10.1002/jor.20653

[15] Kutty S Schneider P Faris P Reliability and predictability of the centre-edge angle in the assessment of pincer femoroacetabular impingement. Int Orth (SICOT) 2012; 36: 505–10. 10.1007/s00264-011-1302-y PMC3291778 21720863

[16] Carlisle JC Zebala LP Shia DS Reliability of various observers in determining common radiographic parameters of adult hip structural anatomy. Iowa Orth J 2011; 31: 52–8. PMC3215114 22096420

[17] Montgomery SR Ngo SS Hobson T Trends and demographics in hip arthroscopy in the United States. Arthroscopy 2013; 29: 661–65. 23375668 10.1016/j.arthro.2012.11.005

[18] Siebenrock KA Kalbermatten DF Ganz R Effect of pelvic tilt on acetabular retroversion: a study of pelves from cadavers. Clin Orthop Relat Res 2003; 407: 241–48. 12567152 10.1097/00003086-200302000-00033

[19] Shrout PE Fleiss JL Intraclass correlations: uses in assessing rater reliability. Psychol Bull 1979; 86: 420–8. 18839484 10.1037//0033-2909.86.2.420

[20] Clohisy JC Carlisle JC Trousdale R Radiographic evaluation of the hip has limited reliability. Clin Orthop Relat Res 2009; 467: 666–75. 19048356 10.1007/s11999-008-0626-4 PMC2635468

[21] Bedi A Dolan M Magennis E Computer-assisted modeling of osseous impingement and resection in femoroacetabular impingement. Arthroscopy 2012; 28: 204–10. 22244100 10.1016/j.arthro.2011.11.005

[22] Milone MT Bedi A Poultsides L Novel CT-based three-dimensiona software improves the characterization of cam morphology. Clin Orthop Relat Res 2013; 471: 2484–91. 23361933 10.1007/s11999-013-2809-x PMC3705074

[23] Monazzam S Bomar JD Cidambi K Lateral center-edge angle on conventional radiography and computed tomography. Clin Orthop Relat Res 2013; 471: 2233–37. 23070664 10.1007/s11999-012-2651-6 PMC3676615

[24] Tannast M Zheng G Anderegg C Tilt and rotation correction of acetabular version on pelvic radiographs. Clin Orthop Relat Res 2005; 438: 182–90. 16131889 10.1097/01.blo.0000167669.26068.c5

